# Cultural adaptations and cultural factors in EBI implementation with Latinx communities

**DOI:** 10.3389/fpubh.2023.1007328

**Published:** 2023-03-21

**Authors:** Felipe González Castro, Cady Berkel, Dana R. Epstein

**Affiliations:** ^1^Center for Health Promotion and Disease Prevention, Edson College of Nursing and Health Innovation, Arizona State University, Phoenix, AZ, United States; ^2^College of Health Solutions, Arizona State University, Phoenix, AZ, United States

**Keywords:** cultural adaptations, cultural factors, mixed methods and research methodology, Latinx (Hispanic), NVivo

## Abstract

**Introduction:**

*Cultural factors* are constructs that capture important life experiences of Latinx/Hispanic individuals, families, and communities. Despite their importance for Latinx communities, Latinx cultural factors have yet to be fully incorporated into the literature of many social, behavioral science, and health service fields, including implementation science. This significant gap in the literature has limited in-depth assessments and a more complete understanding of the cultural life experiences of diverse Latinx community residents. This gap has also stifled the cultural adaptation, dissemination, and implementation of evidence based interventions (EBIs). Addressing this gap can inform the design, dissemination, adoption, implementation, and sustainability of EBIs developed to serve Latinx and other ethnocultural groups.

**Methods:**

Based on a prior Framework Synthesis systematic review of Latinx stress-coping research for the years 2000–2020, our research team conducted a thematic analysis to identify *salient Latinx cultural factors* in this research field. This thematic analysis examined the Discussion sections of 60 quality empirical journal articles previously included into this prior Framework Synthesis literature review. In Part 1, our team conducted an exploratory analysis of potential Latinx cultural factors mentioned in these Discussion sections. In Part 2 we conducted a confirmatory analysis using NVivo 12 for a rigorous confirmatory thematic analysis.

**Results:**

This procedure identified 13 salient Latinx cultural factors mentioned frequently in quality empirical research within the field of Latinx stress-coping research during the years 2000–2020.

**Discussion:**

We defined and examined how these salient Latinx cultural factors can be incorporated into intervention implementation strategies and can be expanded to facilitate EBI implementation within diverse Latinx community settings.

## Introduction

The cultural adaptation of *evidence-based interventions* (EBIs) is now recognized as an important procedure for the effective dissemination and implementation of EBIs that are culturally relevant and acceptable within many Latinx/Hispanic^1^ communities (1). The Latinx population in the US is the largest racial/ethnic population in the nation, with an estimated population in 2021 of 61.3 million, which constitutes 18.9 percent of the total US population of 326.195 million (2).

Cultural adaptations can increase an intervention's capacity to engage participants, also potentially increasing the intervention's effectiveness. Over the past decade, the cultural adaptation of EBIs has been recognized as essential for effective EBI dissemination and implementation within diverse ethnocultural communities (3, 4). Unfortunately, implementation strategies have been almost non-existent that utilize cultural factors to inform the design, dissemination, adoption, implementation, adaptation, and sustainability of EBIs for effective delivery with residents from diverse ethnocultural communities (5, 6). This paper discusses approaches in the *cultural adaptation* of EBIs that can be enhanced by the utilization of cultural factors. A long-term goal of these cultural adaptations is to implement these strategies toward attaining *health equity outcomes* within various Latinx communities (7).

### Historical background on the cultural adaptation of evidence-based interventions

The Fidelity-Adaptation Dilemma emerged in the early 2000's. It juxtaposes two perspectives about the delivery of evidence-based interventions. One argument focused on EBI implementation with *high fidelity* for maintaining its effectiveness in changing targeted intervention outcomes (8). A concern with EBI adaptations was that making intervention changes could erode the intervention's effectiveness. A competing argument was a recognition of the need to make necessary EBI adaptations in response to significant problems encountered in EBI implementation (9). Framed as an either-or proposition, this debate highlighted controversies and consequences of taking one course of action over the other (10). The approach of identifying a “balance,” point among both alternatives emerged as a possible solution, although ultimately it was unsatisfactory for resolving this dilemma.

After two decades of this controversy, the view emerged that fidelity and adaptation are *equally important*. This reframing introduced a more nuanced yet more effective approach for advancing beyond this original “either-or” dichotomy into a more inclusive “both-and” strategy (5, 10, 11). Specifically, defining adaptations as additions to intervention content or structure, rather than simply a lack of fidelity, helped to define which adaptations may be advantageous in promoting cultural fit and in enhancing intervention effectiveness (12, 13).

### Rationale for conducting cultural adaptations

#### History of the emergence of Latinx cultural factors

Reference to “cultural factors” and to “cultural variables” emerged in the early 1970s in an article in 1973 by social psychologists Triandis, Malpass, and Davidson that connected psychology with culture (14). In the 1990s and into the year 2000, Latinx investigators indicated that certain cultural constructs (*cultural concepts, cultural variables, cultural factors*) captured important cultural experiences in the daily lives of many Latinos and Latinas in the US (15, 16). This led to conceptualizing and creating new scales that provided reliable and valid measures of these important cultural constructs. In the drug and alcohol research field, cultural factors believed to capture important life experiences among ethnocultural communities included: (a) acculturation experiences; (b) aspects of stress, coping, and social support; and (c) cultural beliefs and attitudes about drug and alcohol use and misuse (16). Incorporating cultural factors into regression models advanced our understanding of how cultural factors such as *acculturation stress* could influence alcohol and drug use, and how racial and ethnic values, gender norms, and other cultural variables could operate as *moderators* or *mediators* of drug and alcohol use and misuse among various Latinx/Hispanic communities (17).

In 1995, Cuellar and colleagues examined what they called *cognitive referents* of acculturation. They regarded these cultural referents as important constructs for understanding the role of culture among persons of Mexican heritage (18). Constructs that Cuellar and collaborators examined were: familism (*familismo*), fatalism, *machismo, personalismo*, and folk beliefs. Those investigators measured these constructs with new scales which they developed that had sound psychometric properties. In their psychometric analyses, Cuellar and colleagues found that each of these constructs, except *personalismo*, were negatively correlated with *levels of acculturation* to the US society as measured with a unidimensional scale of the acculturation construct. This indicated that these four constructs were more strongly endorsed by Mexican Americans of *lower* acculturation status, that is, persons most actively endorsing and practicing in their daily lives Mexican cultural beliefs, attitudes, behaviors, norms, and other sociocultural activities. Generally these cultural practices have often been associated with beneficial outcomes (e.g., fewer internalizing and externalizing symptoms, lower rates of alcohol use, and improved academic self-efficacy and achievement), when examined in longitudinal research with Mexican American adolescents (19–21).

### Inattention to culture and cultural factors

#### About “culture” broadly conceived

Many definitions of “culture” describe three of its core aspects (22). First, culture consists of a *cognitive schema* or “world view,” shared by a social group and that it is distinct from that of another social group. This schema has also been described as a “subjective culture,” which is, “a cultural group's characteristic way of perceiving its social environment,” and consists of, “attitudes, norms, roles, values, expectancies, and other constructs” [(14), p. 359]. Second, culture consists of well-established community focused *normative beliefs and behaviors* accepted and practiced by members of that social group. Third, this cultural schema is transmitted across generations from elders to youth, so that the norms that define that culture persist across time (22). Implicit and explicit strategies for transmitting these cultural values from parents to children are referred to as *ethnic socialization*, which has been demonstrated to promote multiple positive outcomes among Latinx youth, particularly in the face of discrimination (20). Beyond ethnocultural groups, diverse entities including organizations, also develop social norms, spoken or unspoken, that operate as standards of conduct that govern organizational beliefs and behaviors (22).

#### Missingness of cultural analyses

In 1991 the National Institute on Drug Abuse identified 13 principles of effective drug abuse treatment (23). However, among these 13 principles, *none* mentioned or alluded to *culture* or *cultural factors* as important for effective drug abuse treatment of ethnocultural clients or patients. Similarly, in the field of implementation science, the Expert Recommendations for Implementing Change (ERIC) project introduced 73 strategic recommendations to guide approaches for intervention dissemination and implementation (24). Here also, none of these strategic recommendations mentions or alludes to the utilization of culture or cultural factors in the delivery of EBIs. Given this absence of cultural factors in research, Ramirez Garcia (25) emphasized that a major gap exists in several research fields. This includes the field of dissemination and implementation research.

#### Types of Latinx cultural factors

Traditional cultural factors, the classic cultural factors often mentioned in prior Latinx social and behavioral science research literature include: (a) *familismo*, Latino family closeness and cohesion; (b) *machismo*, traditional Latinx male identity characteristics, both positive and negative; (c) *personalismo*, the importance of warm interpersonal relations; (d) *respeto*, respectful exchanges in interpersonal relations; and (e) *simpatia*, a deferential interpersonal style that strives to maintain harmony in interpersonal relations (6, 26) (see Table 1). Cultural factors also include constructs that describe the process of cultural change among immigrants and others during the *process of acculturation* (27). These include experiences of discrimination (20, 28).

**Table 1:** Major cultural factors in the Latinx research literature.

**Cultural factor**	**Description**
**Multiethnic**	
Acculturation	• Refers to the process of cultural change toward the mainstream culture of a new host society, e.g., in the United States, the American lifestyle
Assimilation	• A change in cultural identity and lifestyle involving a conversion from one's original native culture to a new culture, identity, and lifestyle
Ethnic pride	• A positive attitude and sense of belonging to one's ethnic cultural heritage or native cultural group, e.g., stating that, “I am proud to be a Mexican”
Folk beliefs	• Beliefs in the therapeutic effects of herbal and other natural remedies, including the healing power of spiritual healers
Collectivism-individualism	• Contrasting cultural “worldviews,” involving preferences for an individualistic self-oriented relational style vs. a group-oriented collectivistic relational style
Spirituality	• Beliefs in the influences of God or a higher power, including a strong appreciation and bonding with nature
Traditionalism	• Conservative beliefs and behaviors favoring an adherence to long-establish cultural beliefs and norms about the “correct” way of life and living
**Latinx/Hispanic**	
Acculturation stress	• Involves chronic stress from the challenges and conflicts encountered during the process of cultural changes from one's native culture to a new host culture
Bicultural identity	• The development of a combined identity from living in two cultures, which involves the skills and capabilities for engaging in the languages, activities, and social relations of two distinct societies
Cultural flex	• The skills and capabilities for shifting back and forth in the language, activities, and social relations that exist between two distinct societies
Familismo	• Strong familial orientation, bonding, and devotion to one's family
Machismo	• Traditional Latinx male gender role orientation that emphasizes male dominance as a proper or acceptable form of male identity and conduct
Marianismo	• A traditional Latinx female (Latina) gender role orientation that emphasizes a motherly, nurturant role as well as a demure posture toward males within the household
Personalismo	• The value and preference afforded to personalized attention and courtesy expressed in interpersonal relations
Respeto	• The value of expressing respect and recognition toward persons of higher social position that includes reverence and respect for elders
Simpatia	• A deferential posture toward family members directed at maintaining harmony in family relations that is characterized by a posture of agreeableness, respect, and politeness toward others

Adapted from Castro and Kessler (6).

Table 1 presents important cultural factors mentioned as pan-cultural (multiethnic), and those that are specific to Latinx communities. Castro and Kessler describe other cultural factors of importance within major ethnocultural communities in the US: African American/Black, Asian American and Pacific Islander, Native American/American Indian, and other ethnocultural groups (6).

In summary, despite prior research on cultural factors as constructs that capture the deep structure cultural experiences of Latinx communities, these major cultural constructs have seldom been utilized to guide EBI development and strategic planning in EBI dissemination and implementation. Accordingly, the need exists for guidelines and strategies for incorporating cultural factors into EBI design, dissemination and implementation for delivery with Latinx and other ethnocultural groups in the US: Blacks/African Americans, Asian Americans and Pacific Islanders, and Native Americans/American Indians and Alaska Natives.

### Mixed methods approaches for depth of analysis

Mixed methods research focuses on the integration of qualitative and quantitative data for producing a greater “yield” of information generated from a deep structure analysis that utilizes mixed methods approaches (29), and can yield more comprehensive answers to overarching research questions.

#### Qualitative data

Qualitative data can yield contextual descriptions of complex “real world” situations. This context can inform the development of culturally-relevant EBI adaptations (4), and inform complex process analyses about effective implementation strategies. Typically, qualitative data consist of verbal responses to open ended questions often obtained from individual interviews or focus groups. These rich, complex, nuanced, and informative text narratives can inform the design of culturally relevant EBIs (30). These data include qualitative descriptive observations of interactions or environmental processes, such as socialization practices for teaching children how to respond to discrimination (31). Furthermore, such observational data also includes the identification of an EBI's problematic content or processes that can be rectified with cultural adaptations (32).

#### Quantitative data

Typically, quantitative data are obtained from surveys and questionnaires that capture data using numeric ratings and scales. Generated quantitative (numeric) data, i.e., *measured variables* allow the analysis of correlations between a scaled (measured) cultural factor and numerically transformed data, i.e., *thematic variables* (33). Data analyses that examine associations among measured variables and thematic variables can be conducted using multiple regression model analyses. In summary, whereas *qualitative* data are useful for conducting in-depth, often *exploratory* data analyses, *quantitative* data are useful for conducting *confirmatory* analyses that allow hypothesis testing, and testing a variety of models.

#### Mixed methods data analytic procedures to inform D&I research

The hallmark of mixed methods approaches is the *purposeful integration* of *qualitative* (QUAL) and (QUAN) *quantitative* data, to generate greater yield (explanatory output) from the analysis of these integrated datasets. In these analyses, cultural factors often can be modeled as potential *effect modifiers*, that is, as *moderator variables*, that may change the effects of a *predictor variable* on an *outcome variable* (34). Mixed methods research typically adopts a *pragmatic* approach that is initiated with an *overarching research question*, followed by the planful utilization of mixed methods procedures to yield a more complete answer to that overarching research question (29).

Palinkas and collaborators identified five major reasons for using mixed methods designs in intervention research that focuses on dissemination and implementation issues (35). These reasons include: (a) using quantitative methods to measure intervention and/or implementation outcomes, and qualitative methods to understand process; (b) conducting both exploratory and confirmatory research; (c) examining both intervention content and context; (d) examining consumers' perspectives about the effects of an EBI or other clinical practices, e.g., the views of practitioners and clients; and (e) the capacity to compensate for one type of data analytic method by using another type of data analytic method.

#### Unpacking multidimensional (complex) constructs

Mixed methods can inform the “unpacking” of complex, multidimensional constructs, including several cultural factors. A mixed methods *convergent design* can be used to conduct thematic analyses to reveal underlying dimensions (themes) that define facets of a cultural construct, such as *traditionalism* (36). After conducting a thematic analysis, and utilizing *Scale Coding* (33), investigators can examine associations among the construct's emerging themes.

Using *Scale Coding*, an identified thematic category (scored 1 = present in a case, and 0 = not present) can be transformed into numeric form, i.e., into a *thematic variable* (33), which affords the ability to create a mixed methods correlations matrix to examine select correlations among *measured variables* and transformed *thematic variables* (33, 36). Then, *re-contextualization* involves a return to the analysis of select qualitative text narratives from cases identified by significant correlations within a mixed methods matrix.

### Frameworks for guiding EBI implementation planning and cultural adaptations

In summary, cultural factors can be utilized to inform implementation strategies that can improve EBI implementation among low resource and ethnocultural communities. The incorporation of cultural factors in the cultural adaptation of an original EBI is an important strategy for promoting health equity (37, 38) among diverse communities of color. Cultural adaptation is an important approach that can contribute to increasing the relevance of implementation science for improving the transfer of science to practice for delivery among diverse ethnocultural communities. This approach can build on the rich array of theories, models and frameworks developed under the auspices of implementation science (39). These theories, models, and frameworks (TMFs), constitute organized approaches for guiding adaptation planning (40). Many of these TMFs have undergone revisions to support a health equity focus (41). This provides opportunities for incorporating cultural factors into EBIs to increase the EBI's cultural relevance toward producing effective outcomes when delivered with diverse Latinx community residents.

Across time, dissemination and implementation (D&I) science has brought greater attention to implementation context and strategies (42) in which cultural factors can operate as *contextual factors*, often as moderator variables, of effect modifying conditions. One D&I model that describes the influences of contextual factors is the Practical Robust Implementation Sustainability Model (PRISM) (43). This model can inform various approaches in implementation planning.

Regarding ways to conduct EBI adaptations, experts in the D&I field have recommended the need to document details of the adaptation process as conducted during the process of an EBI's implementation. This documentation consists of clearly describing additions, deletions, and other necessary modifications (42, 44). This careful documentation can inform future implementation protocols as well as generating empirical evidence to refine and expand these TMFs. A methodology for documenting and tracking adaptations to EBIs is the FRAME. Wiltsey Stirman et al. (13) recently expanded the FRAME for more precise documentation and tracking of adaptations and modifications to an original EBI's implementation strategies.

## Materials and methods

### A framework synthesis review of literature

#### Building on prior research

In a prior study, Castro et al. (45) conducted a *Framework Synthesis Review* (46) of the Latinx/Hispanic stress literature for the years 2000–2020. The Framework Synthesis approach utilizes an initial model that is informed and expanded by results from the subsequent framework synthesis review of the literature. The purpose of that prior study was to review two decades of Latinx stress-coping research to inform the development of an expanded and culturally relevant Stress-Coping-Outcomes Model. Our team of four research investigators consisted of two senior research investigators (FC and RC) and two early career investigators (DS and CD).

In that Framework Synthesis Review, we conducted a conventional PRISMA literature review protocol consisting of four steps: (a) *identification*, (b) *screening*, (c) *quality assessment*, and (d) *inclusion* (45). We used PsycInfo and Medline as search engines to identify empirical Latinx stress-coping research studies conducted from the years 2000 to 2020. From this article screening and analysis, our team identified 50 articles from high-impact journals, those having an Impact Factor of 1.5 or higher, for possible inclusion into this systematic review. In this selection process our *quality assessment* of candidate articles consisted of two quality assessment criteria: (a) *Stress Model Relevance*—the requirement that the empirical article examined and described in detail one or more stress-coping model constructs or factors, and (b) *Latinx Cultural Relevance*—that the empirical article utilized a Latinx theoretical framework on culture that examines and describes in detail major cultural aspects of Latinx cultures. For thoroughness in coverage, we conducted an additional screening of select competitive articles from low-impact factor journals. We reviewed these articles using the same review protocol used in our original rigorous screening process. In this manner and based on the review and analysis of 16 candidate articles, our coding team of four investigators included 10 articles that passed our noted two-factor quality assessment.

### Part 1: An exploratory thematic analysis

#### Steps in screening for salient cultural factors

Our team of four investigators conducted an *exploratory* identification of Latinx cultural factors from the Discussion section from each of these 60 empirical journal articles from the field of Latinx stress-coping research. We focused on Discussion sections of these included journal articles (*n* = 60), since Discussion sections typically provide an analysis and interpretation of the empirical study's research outcomes, at times mentioning relevant Latinx cultural factors. Each of our four investigators screened 15 articles from among these 60, to identify candidate cultural factors. We utilized a *Cultural Factor Extraction Form* that our team developed. We then created a Word file of the text narrative of each journal article's Discussion section, and each investigator utilized the Word software program's highlight function to mark a relevant text narrative, typically one to two sentences containing a candidate cultural factor. In follow-up Zoom meetings, we conducted a Roundtable Discussion (33) to consider each candidate cultural factor and included it in this screening analysis if endorsed by all investigators thus reaching consensus on its inclusion.

Results of these analyses were entered into a *Cultural Factor Listing*. From this process we identified 58 constructs/cultural factors. Each of these was “tagged” with the originating journal article's identifier ID number. Under a hierarchical structure, we identified 10 “word stems” as the superordinate term (core construct) that formed the basis of related sub-terms (sub-constructs). For example, the word stem of “acculturation” served as the core construct for the related sub-terms: “acculturation gap,” “acculturation process,” “acculturative stress” and “level of acculturation” (see Table 2).

**Table 2:** Empirically identified cultural factors in Latinx stress-coping-outcomes literature: years 2000–2020.

**Word stems**	**Specific Latinx cultural factors**	**Number of mentions in journal discussion sections**	**Textual passages illustrating salient cultural factors in context**
Acculturation	Level of acculturation	5	• “Culturally relevant questions that remain to be explored in future research are the relationship between acculturation stress and other culturally relevant constructs, such as ***level of acculturation*** and ethnic identity in children.” [(47), p. 222] • “…we did not assess participants' ***level of acculturation*** … future research utilizing self-rated health questionnaires [should] incorporate a measure of acculturation to determine the extent to which (acculturation) influences Hispanic individuals' responses to (other assessed) measures.” [(48), p. 280]
	Acculturation process	5	• “First, there is indeed a link between *acculturation* and *acculturation stress*, but this is an *inverse association* by which those low in the ***acculturation process*** report more stress.” [(49), p. 1442] • “It is critical to remember that the Latinx/Hispanic population is *highly heterogeneous*, and that measuring and conceptualizing cultural phenomenon (e.g., values and ideals of appearance, cultural norms, the ***acculturation process***) are challenging and complex.” [(50), p. 199]
	Acculturation stress/stressor	9	• “….. ***acculturation stress*** increased the risk of PPC [physical-psychiatric comorbidity] for both women and men… This result, along with other studies, demonstrates the powerful effect immigration-related stressors can have on physical and mental functioning.” [(51), p. 209] • “…. the added emotional burden of ***acculturation stress*** on less acculturated—to the US culture— Hispanics may significantly disrupt their family life and further fracture their connection with the home culture.” (52) (p. 520)
Bicultural	Bicultural stress	5	• “The measure of ***bicultural stress*** used in this study captures the degree to which bicultural environments are perceived as problematic for the adolescent. Adapting to a bicultural environment and learning to balance the demands of family, school, and social contexts in a new country represents a long-term, experiential learning curve that probably occurs over the course of adolescence and perhaps even into adulthood.” [(53), p. 10]^*^ • “Exposure to both heritage and US cultures may create ***bicultural stress*** (pressures to balance the two cultural streams) and may increase perceptions of discriminatory actions and of an unfavorable context of reception.” (54) (p. 8).^*^
Cultural	Sociocultural stress/stressor	5	• “As youth experience ***sociocultural stressors***, which may undermine youths' sense of belonging to the US, they may experience a dissonance that eliminates the protection that US identity belonging affords.” [(55), p. 574]. • “Consistent with prior studies … ***sociocultural stress***, … positively predicted alcohol initiation.” [(55), p. 574]
Discrimination	Perceived discrimination	7	• “Moreover, ***perceived discrimination***, a particular factor in *acculturative stress*, has been shown to have a strong relationship to psychological and sociocultural adaptation in cross-cultural samples of adolescents.” [(47), p. 223] • “…. nonimmigrant groups may report higher stress attributed to ***perceived discrimination*** than immigrant groups …. whereas immigration-related stress may be more salient for immigrant children. [(47), p. 222]
	Discrimination stress	8	• “For males, …. ***discrimination stress*** was associated with both suicidal thoughts and self-harm behavior. For females, Family Drug Stress was associated with suicidal thoughts. Acculturation Gap Stress, Family Drug Stress, and Immigration Stress were all significantly associated with self-harm behaviors.” [(17), p. 6]^*^ • “Results of the current study found that ***discrimination stress*** was predictive of Hispanic male suicidal ideation. Prior research has confirmed that racial or ethnic discrimination can create a source of daily stress for Hispanic adolescents. Again, it is unclear why this was predictive among Hispanic males but not Hispanic females.” [(17), p. 8]^*^
Ethnic	Ethnic identity	7	• “A secure ***ethnic identity*** provides ethnic minorities with a sense of belonging that contributes to psychological well-being …. No research to date has evaluated how *acculturative stress* may affect the development of a strong sense of ethnic identity in Hispanic youth.” [(47), p. 222] • “…. ***ethnic identity*** appears to have a significant positive relationship with *thriving*. The secondary main predictor was internal assets. Internal assets are values or competencies that youth have internalized, such as achievement motivation, honesty, integrity, and self-esteem.” [(56), p. 518]
Family	Familism/(familismo)	4	• “The increased levels of depression among Latino caregivers may be related to the Hispanic values of ***familismo*** and *dignidad*. These caregivers strongly believe it is their duty and honor to care for their loved ones in the home … [although] the challenges of caring for a person with [a major disability] especially in advanced stages, [and with] resources within a home …. not adequate to provide the care that may be desired… caregivers may feel they are unable to fulfill their family duties.” [(57), p. 677] • “…. given the importance of ***familismo***, which stresses the centrality of family and adherence to familial values and norms …. the use of substances by parents may be especially relevant to Hispanic adolescent substance use.” [(58), p. 10]^*^
	Family support	6	• “…. females may be particularly vulnerable to disruptions in ***family support***, communication, attachment, and other protective factors. Among the most powerful family risk factors is exposure to drug use behaviors that can increase the risk of adolescent suicidal ideation.” [(17), p. 8]^*^ • “There is the perception that Latinos get lots of help and social support from family members. In reality there is an “over idealization” of the Latino ***family support*** system as many caregivers find themselves as the sole provider.” [(57), p. 679]
Immigration	Immigration status	4	• “Researchers in future studies are encouraged to assess the impact of ***immigration status*** (e.g., immigrant, second-generation) directly on experiences of *acculturative stress* (i.e., involving immigration-related or perceived discrimination).” [(47), p. 222] • “The findings from our study suggest that well-being outcomes for Mexican immigrants and Mexican Americans living in the United States are influenced by a number of factors, including successful employment (income), gender, age, and acculturation, and that years of residency and ***immigration status*** are not significant predictors of well-being.” [(59), p. 461]
	Immigration stress	7	• “Results from this study also indicate that ***immigration stress*** was predictive of self-harm behavior among Hispanic females. Prior research indicates that immigration stress plays a significant role in suicidal ideation. Indeed, immigrant adolescents are at greater risk for a number of mental health and school-related concerns.” [(17), p. 7]^*^ • “…. women may report less ***immigration stress*** because they may perceive advancement in social status after immigrating due to gains in independence and decision-making ability …. Another reason the link between immigration stress and alcohol use may have been stronger among men is that men are more likely to use alcohol to cope with stress while women are more likely to develop internalizing symptoms.” [(60), p. 10]^*^
Traditionalism	Traditional values	8	• “Espousal of traditional familial values protected students from the otherwise strong relationship between depressive symptoms and high acculturative stress.” [(61), p. 859–61] • “Low-income, recent Latino immigrants typically experience limited and difficult work opportunities and living conditions …. among low-income relatively recent immigrants, documented status does not mitigate the contribution of family separation and lower levels of acculturation (i.e., lack of language skills and preference for ***traditional values***) to the stress immigrants experience in relation to their difficult everyday existence.” [(62), p. 12]^*^

* Indicates the reported page numbers as those in the available pdf document.

### Part 2: A confirmatory thematic analysis

#### Rigorous thematic analysis using NVivo 12

Theme identification has been described as, “One of the most fundamental tasks of qualitative research” [(63), p. 85]. The identification of themes has also been described as a key step in unpacking the factorially complex construct of “culture.” Based on our Part 1 exploratory analyses we utilized NVivo 12 Pro to conduct a rigorous confirmatory thematic analysis to identify salient Latinx cultural factors using our original 10 word stems (i.e., acculturation, bicultural, caregiver, cultural, discrimination, ethnic, family, identity, immigration, and traditionalism).

As examined across textual contents in the 60 Discussion sections we conducted a textual search of candidate cultural factors identified in our exploratory analyses, also indicating their frequency of mention (occurrence). We scanned these Discussion sections using a *Key-Word-in-Context* (KWIC) procedure to identify *salient Latinx cultural factors* from the Latinx stress-coping literature across the years 2000–2020. In this casewise analysis of these Discussion sections, we regarded each journal's discussion section as an *independent observation*. Here, the *unit of analysis*, the “case,” was the journal article based on our text review of its Discussion section. Finally, we defined the occurrence of a *theme* as the mention (occurrence) of a given cultural factor in four of these journals, thus constituting a “logical thread” that was mentioned across four or more of these independent journal observations (63). In summary, in this confirmatory analysis, we utilized the NVivo 12 *Word Search Wizard* to scan each journal article's Discussion section to detect the occurrences (mentions) of each of our candidate cultural factors, to identify the cultural factors mentioned across independent observations, i.e., journals, also indicating their frequency of mention. The cut-point of at least four mentions across different journals was used to identify the occurrence of a theme, which indicates the salience of that candidate cultural factor. The identification of that salient Latinx cultural factor constitutes a logical thread occurring across two decades within the Latinx stress-coping literature.

## Results

As noted, we conducted a rigorous approach to confirm the prior exploratory analysis of cultural factors. We used the NVivo 12 software program to conduct a Key-Word-in-Context (KWIC). In this analysis we identified 13 themes that constitute *salient Latinx cultural factors* appearing in the Latinx stress-coping research literature for the years 2000–2020 (see Table 2).

### The salient Latinx cultural factors

Table 2 presents these 13 specific Latinx cultural factors (see column 2). Eight of the our original 10 stem terms, i.e. “acculturation,” “bicultural” etc., are presented in column 1, as the stem terms were used to identify these Latinx cultural factors. These eight stem terms revealed a total of *13 specific Latinx cultural factors*, which could be defined as *salient Latinx cultural factors* (see Table 2, column 2). Presented in column 3 is the number of different journal articles mentioning cultural factors in their Discussion section. Finally, column 4 presents illustrative textual passages from relevant journal article sections that illustrate in context the application of each salient Latinx cultural factor in the research study in which this factor appeared. Here we include two passages for a broader illustrative context.

#### Acculturation: Level of acculturation, acculturation process, acculturation stress

From the broader construct of acculturation, three related salient Latinx cultural factors emerged: “level of acculturation,” “acculturation process,” and “acculturation stress/stressors.” Relevant contextual quotations included in column 4 provide meaning and context for understanding and assessing various aspects of acculturation among diverse Latinx communities. The heterogeneity of the Latinx/Hispanic population is also indicated, suggesting the utility of conducting in-depth analyses with distinct Latinx population sectors by *level of acculturation* (low acculturation, bicultural, high acculturation).

#### Bicultural stress

The construct of bicultural stress refers to the daily experiences of US-born and immigrant Latinx individuals who often struggle to succeed within two distinct cultural environments, i.e., the American and Latinx cultures. These passages reveal that efforts at adaptation to a new culture or setting are often stressful, resulting from exposures to conflicting sociocultural stressors, including discrimination, and structural barriers to social and economic mobility (53).

#### Sociocultural stress/stressors

External events (stressors) and the tension they produce (stress) can produce acute and chronic distress followed by mild-to-severe psychological symptoms that can develop into diagnosable psychiatric disorders such as Anxiety and Depressive Disorders, as well as the Drug and Alcohol Abuse Disorders (51, 64).

#### Perceived discrimination and discrimination stress

Discrimination is often a stressful experience for Latinos and Latinas, when experienced at any of several developmental stages of life. Certain Latinx groups often experience greater exposure to discrimination based on their acculturation status and features of their personal identity. These situations can impede normal youth development and identity formation. Among some Latinx individuals, discrimination may lead to suicidal ideation and other forms of self-harm (17).

#### Ethnic identity

Developing a strong and secure ethnic, gender, and other forms of personal identity can contribute to a sense of belonging and well-being. Some research reports a positive association between ethnic identity formation and thriving (51, 56).

#### Famililism/familismol and family support

A strong sense of familism (*familismo*) is often regarded as a personal asset. The context in which familism and family social supports occur often influences a youth's family identification, belongingness, and sense of well-being. However, among some Latinx caretakers of a disabled family member, Latinx family values involving *familismo* and *dignidad* (dignity), coupled with insufficient resources, can produce stressful family conflicts. Substance use by some caretakers to cope with the stressors of caretaking may prompt substance use among adolescent members of the family (58). While *familismo* is prevalent among many Latinx families, this broad view has been questioned, referring to *familismo* as an “over idealization” (57). This can occur among some Latinx caretakers for whom traditional Latinx family expectations and ascribed caretaker roles create burdens for a single family member who is expected to serve as the sole family caretaker (57).

#### Immigration status and immigration stress

Research shows that in coming to the US some Latinas and young adults gain social status, independence, and decision-making abilities, which may empower them for adapting effectively within the US culture (60). Stress experiences among Latinx individuals can vary in relation to gender and to immigration status. Various gender patterns have emerged involving alcohol use among immigrant Latinas in response to multiple sociocultural stressors. Among some Latinx adolescents, immigration stress can prompt suicidal ideation particularly among adolescent Latinas (females) (17).

#### Traditionalism: Traditional family values

Among Latinx young adults, in some families traditional Latinx family values involving strict parental expectations encourage adult children to remain close to the family and to adhere to restrictive traditional family rules (61). Conversely, traditional Latinx family values in everyday life can also exert a positive effect on a family members' health and well-being. Within this context, the *intersectionality* of *traditional family values* with gender, low level of acculturation, immigrant status, and other sociocultural variables can exert complex beneficial and adverse influences on a family member's health and well-being (62).

## Discussion

In comparing the classic Latinx cultural factors presented in Table 1, with the empirically identified Latinx cultural factors in Table 2, some concordance appears in the joint identification of four cultural factors: acculturation, acculturation stress, *familismo*, and traditionalism (traditional family values). By contrast, other empirically identified cultural factors involve various specific types of stress: bicultural stress, sociocultural stress, discrimination stress, and immigration stress. Future studies that examine other areas of research such as family systems research may reveal the salience of several of the traditional Latinx cultural factors which include: *familismo, machismo, personalismo, respeto*, and *simpatia*. Those analyses may identify other important Latinx cultural factors that capture other deeply rooted and important aspects of Latinx cultures.

### Implementation strategies using Latinx cultural factors

#### Building partnerships and creating structural changes for implementation readiness

There is a growing recognition of the importance of collaborative partnerships between researchers and community-based organizations to inform the development and enhance the implementation of culturally responsive EBIs. Community-Based Participatory Research (CBPR) approaches (65) are now regarded as essential procedures for EBI adoption, adaptations, dissemination, and implementation.

A study by Orengo-Aguayo et al. established a university-community partnership for the dissemination and implementation of Trauma-Focused Cognitive Behavior Therapy (TF-CBT) for delivery in three low-resource settings. These interventions and settings were: (a) the delivery of telehealth in a rural community in South Carolina, (b) the delivery of this TF-CBT in the island of Puerto Rico in the aftermath of Hurricane Maria, and (c) building the local infrastructure and workforce for delivering the TF-CBT within impoverished war-ravaged communities within the country of El Salvador (66).

This international translation study that was conducted in El Salvador featured the traditional Latinx cultural factor of *personalismo*, a relational style that emphasizes “personal attention and courtesy in interpersonal relations” (15, 18) (see Table 1). Applying *personalismo* involves relationship building for engaging in intervention activities that facilitate open discussions and mutual collaborations. Among traditional Latinx communities, the act of social engagement observes a Latinx-Hispanic cultural norm and expectation of *personalismo* in customary actions that should precede collaboration in a collective activity.

These investigators recognized the value of using an implementation framework, such as the EPIS (67), while also asserting that these frameworks can be, “extremely limited in terms of contextual adaptabilities” [(66), p. 1172]. These investigators emphasized that D&I research investigators must *listen* to local community stakeholders to truly *hear* their concerns. They also asserted that such listening can inform the development and implementation of, “sweeping solutions to many of the biggest challenges currently facing dissemination and implementation science within low-resource settings” [(66), p. 1172].

In a study by Hirchak and collaborators, university investigators in the Southwestern United States established a strong partnership with a large American Indian/Native American community (68). This *Tribal-University Partnership* fostered community engagement activities during the pre-implementation stage by engaging in active partnerships with residents from this indigenous community. This pilot study consisted of three intervention adaptations aimed at incorporating “cultural re-centering” in an adapted version of the original EBI. Using an Interactive Systems Framework they examined *facilitators* and *barriers* encountered in planning for recruitment, training, and sustainability, in their approach to capacity building which focused on three areas. These areas are: (a) implementation and evaluation, (b) training and technical support provided to tribal residents serving as study collaborators, and (c) generating new data to inform the dissemination of new scientific evidence. Generally these intervention projects focused on building capacity to create implementation delivery infrastructures within these low-resource communities. This approach could be enhanced by incorporating specific cultural factors such as: collectivism-individualism, familismo, traditionalism, personalismo, respeto, and simpatia (see Table 1).

#### Familism and familial factors across the globe

Although research on *familismo* emerged in sociological research conducted with Latinx families, there exists a cross cultural recognition regarding the importance of *familismo* among many world cultures. Williams and collaborators conducted an international implementation project in China's rural Hunan Province. They conducted a culturally adapted nursing intervention to improve medication adherence among people living with AIDS (PLWA) (3). Investigators established an adaptation team in partnership with a local Community Advisory Board. This partnership included persons living with AIDS and local healthcare workers. Cultural issues encountered included: (a) the high value and importance of the family (high *familismo*) within rural Chinese communities; (b) *stigma* as a barrier to treatment participation based on the strong Chinese value and cultural factor of “saving face,” which emphasizes avoiding behaviors that bring shame to one's family, and the importance of preserving family honor; (c) *travel barriers* existing within the rural Hunan province; and (d) the *low level of knowledge* about HIV/AIDS among many residents of these rural Chinese communities.

From this study investigators offered six recommendations for conducting cultural adaptations in a low resource community: (a) involve stakeholders from the beginning; (b) throughout the project, conduct a needs assessment that involves continual assessments; (c) evaluate the original intervention for necessary adaptations to intervention implementation; (d) identify mismatches in intervention components and activities with local culture beliefs and values; (e) identify sources of fidelity-fit tensions; and (f) throughout the project document the process of adaptation (3).

Iguchi et al. conducted a cultural adaptation of a Family-Based Treatment (FBT) for anorexia nervosa for implementation in Japan (69). Investigators reported on systemic cultural barriers encountered in this implementation. Given the strong value of *familismo* within Japanese culture, especially among families who observe traditional gender roles, investigators report that often clinical staff would exclude a youth's parents from participating in the treatment process. Investigators regarded this exclusion as a means of inadvertently disempowering parents and distancing them from a direct role in caring for their child's anorexic nervosa (69). This emerged as a significant *cultural mismatch* and *barrier* to the implementation of a Family-Based Treatment for anorexia nervosa in Japan.

Within this context, the investigator needed to consider that traditional Japanese cultural norms and gender role expectations dictate that Japanese mothers are expected to serve as their child's caretaker, whereas Japanese fathers are expected to be uninvolved with these caretaker duties. These traditional culture gender role expectations needed to be reframed to encourage parental participation. Accordingly, in their cultural adaptation, investigators invited the father to participate in accord with their ascribed role as head of the household. The father was encouraged to attend educational sessions designed to empower parents and to involve them jointly in their child's care. Investigators report that these reframed cultural adaptations improved the delivery of this child-focused anorexia treatment intervention as implemented in Japan.

#### Summary of culturally responsive implementation strategies for cultural adaptation

These studies illustrate some common strategies for incorporating cultural factors into intervention adaptations in the service of intervention dissemination and implementation with diverse ethnocultural groups. These strategies are: (a) from the beginning, *establishing a collaborative partnership* with community stakeholders by incorporating CBPR principles (3, 66); (b) *building community resources* to promote implementation readiness such as training in intervention delivery (66, 68); (c) conducting a *pre-implementation assessment* that includes evaluating intervention contents to rectify *cultural mismatches* and to increase the intervention's cultural acceptability among local community residents (68, 69); (d) conducting a stage-wise process of implementation activities that support drivers of the implementation process; (e) monitoring implementation progress by documenting adaptation changes and reasons for them; and (f) engaging in sustainability planning to ensure intervention continuity.

### Integrating cultural factors into implementation strategies

From the 73 identified implementation strategies presented by the Expert Recommendations for Implementation Change (ERIC) project, Table 3 presents six selected implementation strategies expanded to illustrate how Latinx cultural factors may be integrated into some of these 73 recommendations. For each of six implementation strategies presented, the descriptive column titled, “Possible Expansion with Cultural Factors” presents the original journal article text as supplemented with a cultural adaptation using certain Latinx cultural factors. These culturally adapted extensions are indicated in italic bold face font.

**Table 3:** Selected ERIC implementation strategies as can be expanded with cultural factors.

**Implementation strategy**	**Possible expansion with cultural factors**
1. Develop academic partnerships	• Partner with a university or academic unit for the purpose of sharing training and bringing research skills to an implementation project. ***Intervention project staff can invite the participation of Latinx academic scholars*** **who with their disciplinary expertise** ***can describe and explain how cultural factors can aid in tailoring the intervention for greater cultural relevance for community residents from the major Latinx acculturation sectors (low acculturation, bicultural, high acculturation)***.
2. Intervene with patients/consumers to enhance uptake and adherence	• Develop strategies with ***Latinx*** patients ***of different levels of acculturation*** to encourage and problem solve around adherence ***to an intervention by describing and explaining the cultural factors that are most salient and relevant for residents of the local community***.
3. Invoke patients/consumers and family members	• Engage or include patients/consumers and families ***from local Latinx community neighborhoods*** in the implementation effort ***this includes the application of select Latinx cultural factors***.
4. Obtain and use patients/consumers and family feedback	• Develop strategies to increase patient/consumer and family feedback on the implementation effort ***that is designed with cultural flexibility for best acceptability to major acculturation sectors (low acculturated, bicultural, high acculturated) who are residents of the local Latinx community***.
5. Promote adaptability	• Identify the ways a clinical intervention can be tailored to meet the local needs and clarify which elements of the innovation must be maintained to preserve fidelity, **and** ***what must be modified or new modules added to increase the intervention's cultural relevance for each of the major cultural sectors from the local Latinx community***.
6. Tailor strategies	• Tailor the implementation strategies to address barriers and leverage facilitators that were identified through earlier data collection, ***to examine the relevance and application of certain relevant Latinx cultural factors***.

Adapted from Powell et al. (24).

For example, one strategy is developing university or academic partnerships for, “sharing training and bringing research skills to an important project.” A cultural expansion of this strategy could add that, “*Intervention project staff can invite the participation of Latinx academic scholars*” to provide their disciplinary expertise to “*describe and explain how cultural factors can aid in tailoring the intervention for greater cultural relevance for community residents from the major Latinx acculturation sectors (low acculturation, bicultural, high acculturation)*.” In a similar manner, efforts can promote the acceptability of an intervention by clarifying “which elements of the intervention must be maintained to preserve fidelity.” This strategy can be expanded by determining also “*what must be modified or new modules added to increase the intervention's cultural relevance for each of the major cultural sectors from the local Latinx community.”* This addition emphasizes an approach to cultural adaptation from the perspective of a resolution of the Fidelity-Adaptation Dilemma described earlier. This also involves the need to address the within-population heterogeneity in Latinx populations, by attending to major acculturation sectors that exist among the residents of the local community.

### Contributions to the field

Our study may be one of the first to conduct an empirically based identification of the most *salient Latinx cultural factors* in the literature from the stress-coping research field based on quality research studies published in the years 2000–2020. We also advance beyond other descriptions of Latinx cultural factors by providing specific contexts that can aid in understanding the role of these salient cultural factors in EBI enhancement and fit, and for delivery with Latinx patients/consumers. This may facilitate EBI implementation within diverse Latinx community settings.

Our study also argues that the field of implementation science has not incorporated cultural factors generally, and Latinx cultural factors in particular, as components that can improve implementation strategies in dissemination and implementation research. Although other fields also lack such a focus on cultural factors, given the importance of promoting health equity (38) among ethnocultural groups in the US, we encourage implementation scientists to explore the utilization of various cultural factors to inform the strategic development and adaptation of various EBIs for their effective implementation especially with ethnocultural groups and communities.

### Limitations

Based on the present review's focus on the field of Latinx stress-coping research, our study identified the most salient Latinx cultural factors from that field. We also describe the effects of these salient Latinx cultural factors within the context of stress-coping research. A similar review from literature in a significantly different Latinx research field may produce themes and related cultural factors that differ from those identified in the present study. By contrast, some of the more salient identified themes in our review, such as “acculturation,” “discrimination,” and “immigration” might still emerge in reviews conducted with other research areas.

### Future directions

Future research can further describe and apply Latinx cultural factors to assess their effects in developing a secure ethnic identity toward increasing the efficacy of an intervention such as Trauma-Focused Cognitive Behavioral Therapy. As we promote the effective reach, dissemination, implementation, and sustainability of EBIs within diverse Latinx communities, implementation scientists are encouraged to discover, examine, and apply implementation facilitators and strategies as supplemented by Latinx cultural factors in EBI implementation within diverse Latinx community settings.

## Conclusions

As we have described and illustrated, Latinx cultural factors are rich constructs that can add depth of cultural description and analysis for designing EBIs, and for their dissemination and implementation within various Latinx communities. A greater understanding of contextual aspects introduced by specific cultural factors, such as *levels of acculturation* and *Latinx traditional family values* may reduce or eliminate implementation barriers and inform intervention implementation. In the past, given that Latinx cultural factors received little coverage and utilization in the field of implementation science, implementation scientists are encouraged to better understand the use of Latinx cultural factors, to inform more effective dissemination and implementation strategies. This can enhance an intervention's acceptability and effectiveness and improve its implementation within diverse Latinx and other communities of color.

## Data availability statement

The raw data supporting the conclusions of this article will be made available by the authors, without undue reservation.

## Author contributions

FC conceptualized this study, conducted the NVivo qualitative data analyses with support from his research assistants, and prepared the initial draft of this manuscript. CB contributed to the review of literature, editing, and introduced questions and issues for consideration. DE contributed editorial comments and raised issues and questions to improve this manuscript. All authors contributed to the article and approved the submitted version.

## Footnotes

^1^In this article we use the terms “Hispanic” and “Latinx” synonymously. The term Latinx refers to Latino men (Latinos) and women (Latinas) with the general term Latinx serving as a non-gendered term. Presently, among Latinx/Hispanic scholars there is no consensus in preference for the use of these two terms. The US Census uses the term “Hispanic” to refer to, “people whose origin is Mexican, Puerto Rican, Cuban, Spanish-speaking Central and American countries, or other Hispanic/Latino regardless of race” (2).
